# Supplementary material for: Impact of a school-based water and hygiene intervention on child health and school attendance in Addis Ababa, Ethiopia: a cluster-randomised controlled trial
Source: BMC Med. 2024 Sep 2;22:348. doi: 10.1186/s12916-024-03558-x (PMC11367772; doi:10.1186/s12916-024-03558-x)

Additional file 1: Project WISE infrastructure and delivery

Project WISE infrastructure components were delivered to meet *Splash* target ratios (full details of programme standards can be found at https://splash-program-standards.screenstepslive.com):

- 1 drinking tap per 75 users (including adults/staff), based on the single largest shift population at a campus.
- 1 handwashing tap per 75 users (including adults/staff), based on the single largest shift population at a campus.
- A minimum of 9 L/person/day of water storage capacity, based on the total population of users at a school campus. In Addis Ababa, the target is for each school to have 3 days of water storage supply available.

Project WISE intervention schools were provided with water storage tanks to meet storage capacity standards (e.g., Figure S1, left) and water filtration systems for to supply drinking water stations (e.g., Figure S1, right), meeting the minimum ratio for drinking taps to users (1:75) and the minimum acceptable flow rate per tap (3 L/min). Water quality is tested pre- and post-implementation and routinely throughout the monitoring period.

Figure S1. Water storage tanks (left) and water filtration system (right) installed in study schools.

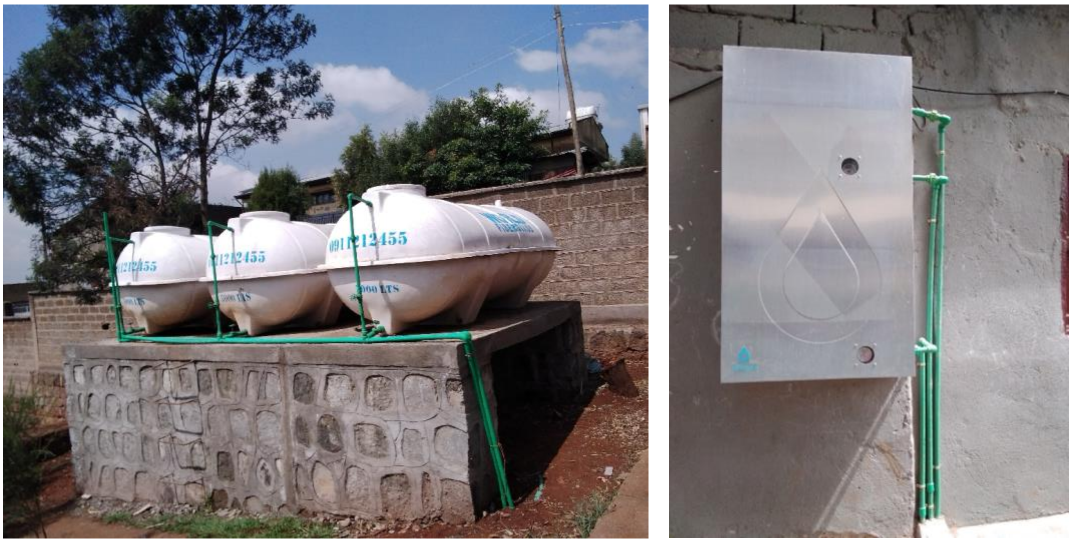

Drinking water and handwashing stations are made from durable fade-resistant HDPE with recycled material, with different colours and basin depths for handwashing and drinking water to promote separation of behaviours and prevent drinking unfiltered water from handwashing stations. An optional riser at the base is intended to allow the station to serve children of various ages, and stations have smooth surfaces with rounded corners for ease of cleaning.

Drinking water stations (Figure S2) had two bottle filler taps per station and a bubbler tap, and were installed in easily visible and accessible locations for the student population, which can include: near

play areas, classroom buildings, or the feeding area. Stations were installed on impervious surfaces such as flat stones or concrete, with a concrete pad provided if no suitable surface was available, and with suitable drainage.

Figure S2. Drinking water stations installed in study schools.

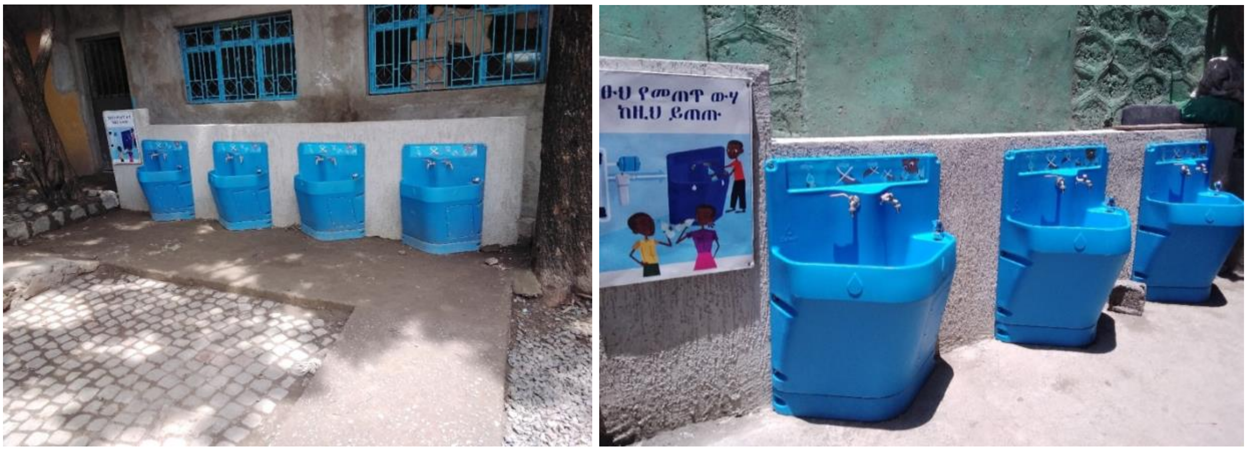

Handwashing stations (Figure S3) had two taps per station, and the sides angled at 45 degrees were designed to promote improved hygiene behaviour through face-to-face handwashing. Handwashing stations were installed to meet targets of at least one handwashing tap for every three toilet stalls and/or urinal spaces, located within 5 metres of the sanitation facility, and at least one handwashing station near the feeding area of the school. Stations were also installed on impervious surfaces such as flat stones or concrete, with a concrete pad provided if no suitable surface was available, and with suitable drainage.

Figure S3. Handwashing stations installed in study schools.

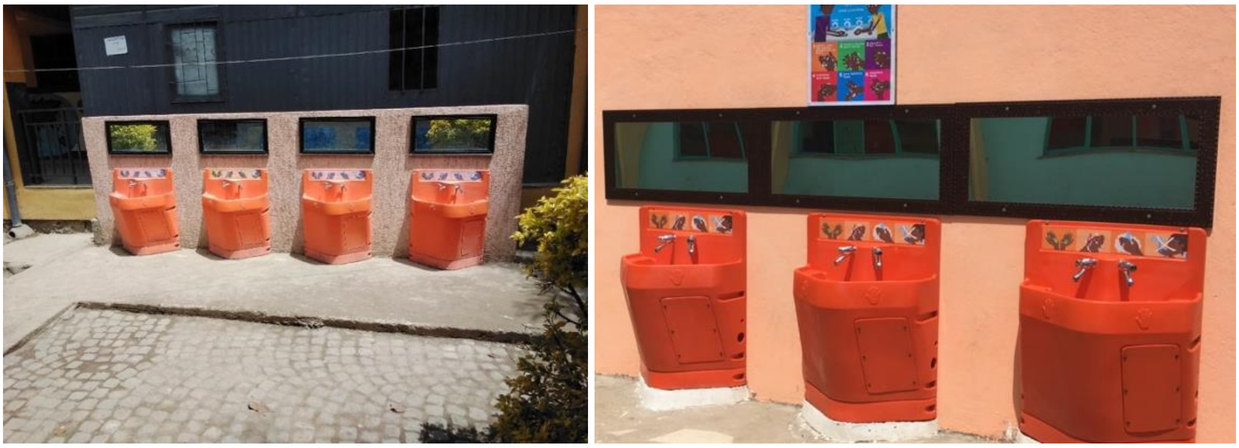

Mirrors made of a shatter-proof material or with a protective clear cover (acrylic) were installed above every handwashing station (both new and existing stations) at an appropriate angle for children. Posters and signs at both drinking water and handwashing stations (Figure S4) provided environmental cues for behaviour and instruction on handwashing technique and correct use of facilities.

Figure S4. Posters and signage at drinking water stations (left and centre) and handwashing stations (right) installed in study schools.

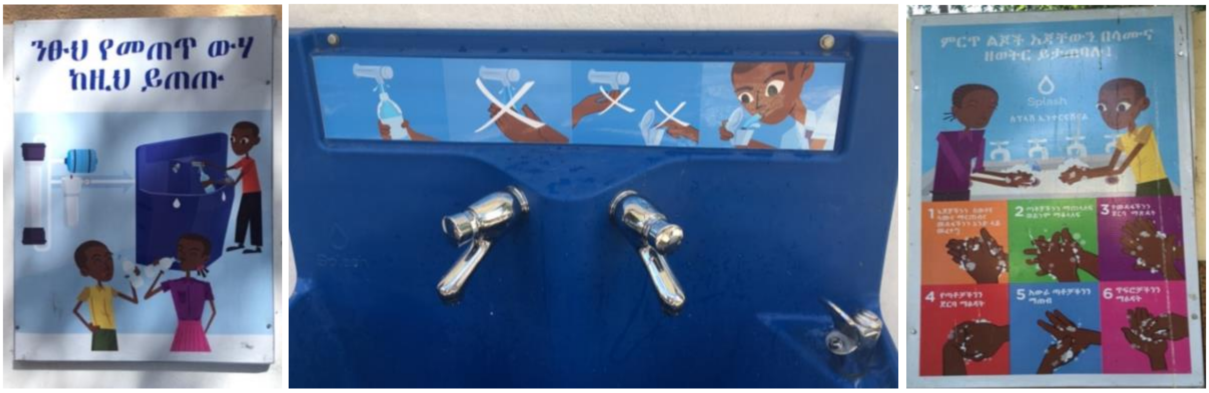

The sanitation components of Project WISE (not delivered until after study completion) were planned to meet target ratios of 1 sanitation fixture per 75 male students, and 1 sanitation fixture per 70 female students. Facilities were to be gender-segregated, separate for students and teachers, wheelchair-accessible, well-lit and ventilated, able to close and lock from the inside, with adequate lighting in the stall and hallway, and a water tap and bucket and waste bin for menstrual hygiene management.

The water and hygiene infrastructure improvements (along with core training modules) were delivered to all 30 intervention schools by January 2022. No control school received any intervention component until after study completion. Intervention and control schools were distributed over a wide area across Addis Ababa (Figure S5).

Figure S5. Approximate locations of 30 intervention (blue) and 30 control (orange) schools enrolled in the WISE trial across Addis Ababa, Ethiopia

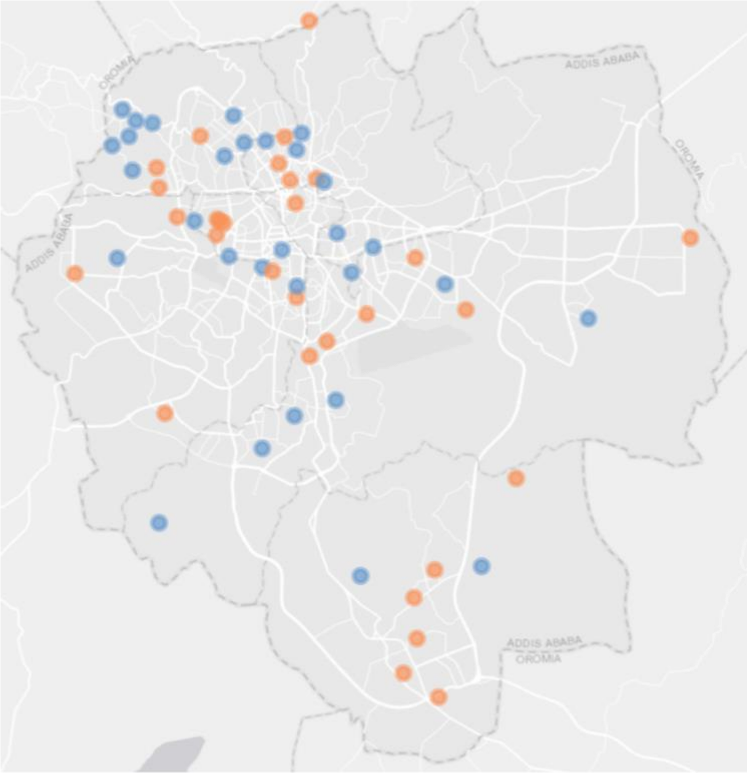

Supplement: Supplementary file 1 — Additional file 1: Project WISE infrastructure and delivery, text accompanying Figures S1–S5. FigS1–Water storage tanks (left) and water filtration system (right) installed in study schools. FigS2–Drinking water stations installed in study schools. FigS3–Handwashing stations installed in study schools. FigS4–Posters and signage at drinking water stations (left and centre) and handwashing stations (right) installed in study schools. FigS5–Approximate locations of 30 intervention (blue) and 30 control (orange) schools enrolled in the WISE trial across Addis Ababa, Ethiopia. [file 12916_2024_3558_MOESM1_ESM.pdf]
