# Supplementary material for: Impact of a school-based water and hygiene intervention on child health and school attendance in Addis Ababa, Ethiopia: a cluster-randomised controlled trial
Source: BMC Med. 2024 Sep 2;22:348. doi: 10.1186/s12916-024-03558-x (PMC11367772; doi:10.1186/s12916-024-03558-x)
Supplement: Supplementary file 2 — Additional file 2. Tables S1–S5. Table S1–Number of participants and observations included in assessment of each outcome at each time-point. Table S2–Intervention effects on primary and secondary outcomes across three models with various adjustments. Table S3–Analysis of mean differences for primary and secondary repeated binary outcomes aggregated at the pupil level. Table S4–Intervention effects on other outcomes. Table S5–Sensitivity analyses. [file 12916_2024_3558_MOESM2_ESM.pdf]

Additional file 2: Supplementary tables

Table S1. Number of participants and observations included in assessment of each outcome at each time-point.

	Follow-up 1		Follow-up 2		Follow-up 3		Final follow-up		Total participants		Total observations	
Outcome	Control	Intervention	Control	Intervention	Control	Intervention	Control	Intervention	Control	Intervention	Control	Intervention
Roll-call absence†	3088	3070	3088	3072	3084	3064	3054	3020	3089	3079	12314	12226
Pupil-reported diarrhoea in past 7 days†	2886	2861	2871	2868	2600	2616	2658	2573	3075	3069	11015	10918
Pupil-reported diarrhoea in past 2 days	2887	2860	2870	2868	2600	2616	2657	2574	3075	3070	11014	10918
Pupil-reported respiratory illness in past 7 days†	2882	2855	2870	2865	2598	2616	2653	2573	3075	3070	11003	10909
Pupil-reported respiratory illness in past 2 days	2882	2857	2870	2867	2601	2616	2655	2574	3075	3070	11008	10914
Pupil-reported full-day absence in past week	2887	2862	2871	2868	2602	2617	2658	2575	3075	3070	11018	10922
(total days of reporting; approx. 5 in past week)	(14488)	(14341)	(14392)	(14339)	(13010)	(13085)	(13377)	(12952)			(55267)	(54717)
SDQ-15 total difficulties score							1829	1675	1829	1675	1829	1675
SAMNS-26 total score							545	438	545	438	545	438
MPNS-36 total score							530	406	530	406	530	406

Note: Total observations included in assessments at each follow-up do not always exactly match number of eligible participants in Figure 1 due to circumstances where absence could not be assessed (e.g., sentinel classes not available for data collection on the follow-up day), or pupil refusals for specific questions. †Primary outcomes.

Table S2. Intervention effects on primary and secondary outcomes across three models with various adjustments.

Outcome	Primary analysis (adjusted for stratification variables)		Further adjusted for grade and gender		Further adjusted for sub-city and time-point*	
	aOR (95% CI)	p-value	aOR (95% CI)	p-value	aOR (95% CI)*	p-value
Pupil-reported diarrhoea in past 7 days†	1.15 (0.83, 1.59)	0.39	1.11 (0.82, 1.52)	0.50	0.95 (0.74, 1.24)	0.72
Pupil-reported diarrhoea in past 2 days	1.22 (0.82, 1.83)	0.32	1.19 (0.81, 1.75)	0.39	0.97 (0.71, 1.33)	0.86
Pupil-reported respiratory illness in past 7 days†	0.84 (0.71, 1.00)	0.046	0.83 (0.69, 0.98)	0.031	0.83 (0.70, 0.99)	0.035
Pupil-reported respiratory illness in past 2 days	0.88 (0.73, 1.06)	0.18	0.87 (0.73, 1.05)	0.14	0.88 (0.73, 1.05)	0.16
Roll-call absence†	1.07 (0.83, 1.38)	0.59	1.08 (0.84, 1.40)	0.53	1.05 (0.81, 1.36)	0.71
Pupil-reported full-day absence in past week	1.01 (0.75, 1.36)	0.93	0.99 (0.74, 1.33)	0.97	1.01 (0.76, 1.36)	0.94
	aMD (95% CI)	p-value	aMD (95% CI)	p-value	aMD (95% CI)	p-value
SDQ-15 total difficulties score (0–40)	0.03 (-0.62, 0.68)	0.94	-0.001 (-0.65, 0.64)	1.00	0.03 (-0.59, 0.65)	0.94
SAMNS-26 total score (0–100)	3.32 (0.05, 6.59)	0.046	3.66 (0.29, 7.04)	0.033	3.41 (0.10, 6.72)	0.043
MPNS-36 total score (0–3)	-0.01 (-0.08, 0.06)	0.81	0.004 (-0.06, 0.07)	0.91	0.01 (-0.04, 0.07)	0.64

Note: Analyses include all eligible children with outcome data at the relevant follow-up. For repeated measures, we calculated a proportion of available follow-ups with illness / absent for each participant, and the mean and SD of these proportions across participants are shown. Stratification variables adjusted for in all models are school size (< or ≥1200 pupils) and presence of a kindergarten. All analyses included a random effect for school, and analyses based on repeated measures included an additional random effect for pupil-level clustering and assumed a constant treatment effect across time-points. *Adjustments for time-point made for repeated measures. †Primary outcomes.

Table S3. Analysis of mean differences for primary and secondary repeated binary outcomes aggregated at the pupil level.

	**Control**		**Intervention**		**Intervention effect**		
Outcome	Pupils	Mean prop. of follow-ups with illness (SD)	Pupils	Mean prop. of follow-ups with illness (SD)	aMD (95% CI)	p-value	ICC
Pupil-reported diarrhoea in past 7 days†	3075	0.073 (0.151)	3069	0.083 (0.160)	0.010 (-0.011, 0.031)	0.35	0.062
Pupil-reported diarrhoea in past 2 days	3075	0.042 (0.112)	3070	0.050 (0.127)	0.009 (-0.006, 0.024)	0.25	0.054
Pupil-reported respiratory illness in past 7 days†	3075	0.276 (0.269)	3070	0.248 (0.257)	-0.032 (-0.062, -0.002)	0.038	0.043
Pupil-reported respiratory illness in past 2 days	3075	0.187 (0.230)	3070	0.171 (0.219)	-0.019 (-0.044, 0.006)	0.14	0.039
	Pupils	Mean prop. of follow-ups absent (SD)	Pupils	Mean prop. of follow-ups absent (SD)	aMD (95% CI)	p-value	ICC
Roll-call absence†	3088	0.103 (0.171)	3078	0.106 (0.171)	0.004 (-0.018, 0.027)	0.70	0.056
	Pupils	Mean prop. of school days reported absent (SD)	Pupils	Mean prop. of school days reported absent (SD)	aMD (95% CI)	p-value	ICC
Pupil-reported full-day absence in past week	3075	0.056 (0.090)	3070	0.055 (0.082)	-0.0001 (-0.015, 0.014)	0.99	0.10

Note: Analyses include all eligible children with outcome data at the relevant follow-up. For repeated measures, we calculated a proportion of available follow-ups with illness / absent for each participant, and the mean and SD of these proportions across participants are shown. Analysis was based on the mean differences in these pupil-level proportions between trial arms, including a random effect for school. Analyses were adjusted for stratification variables (school size < or ≥1200 pupils, and presence of a kindergarten). †Primary outcomes.

Table S4. Intervention effects on other outcomes.

	Control		Intervention		Intervention effect		
Outcome	Pupils	Mean prop. of follow-ups reporting absence (SD)	Pupils	Mean prop. of follow-ups reporting absence (SD)	aOR (95% CI)	p-value	ICC
Pupil-reported absence due to illness in past week	3075	0.102 (0.182)	3069	0.093 (0.173)	0.89 (0.75, 1.05)	0.17	0.017
Pupil-reported absence due to diarrhoea in past week	3075	0.008 (0.047)	3069	0.005 (0.040)	0.59 (0.37, 0.93)	0.024	0.054
Pupil-reported absence due to respiratory illness in past week	3075	0.042 (0.117)	3069	0.039 (0.111)	0.90 (0.72, 1.11)	0.31	0.020
	Pupils	Reported illness at final follow-up (%)	Pupils	Reported illness at final follow-up (%)	aOR (95% CI)	p-value	ICC
Pupil-reported earache in past 7 days	2651	54 (2.04)	2571	61 (2.37)	1.17 (0.77, 1.77)	0.46	0.035
Pupil-reported earache in past 2 days	2656	44 (1.66)	2573	48 (1.87)	1.13 (0.74, 1.71)	0.57	0.001
	Pupils	Median (IQR)	Pupils	Median (IQR)	Proportional aOR (95% CI)	p-value	ICC
Subjective wellbeing – smiley faces visual analogue scale (1–5)	2658	4 (3, 5)	2575	4 (3, 5)	0.92 (0.72, 1.17)	0.48	0.055
	Pupils	Mean score (SD)	Pupils	Mean score (SD)	aMD (95% CI)	p-value	ICC
SAMNS-26 subscales (0–100)							
MH preparation and maintenance	549	72.4 (17.6)	443	75.2 (18.9)	2.96 (-0.20, 6.11)	0.067	0.053
Menstrual pain management	559	65.6 (25.9)	452	68.2 (25.0)	2.33 (-1.29, 5.95)	0.21	0.016
Executing stigmatised tasks	562	59.9 (26.5)	453	62.3 (27.3)	2.59 (-2.11, 7.29)	0.28	0.056
MPNS-36 subscales (0–3)							
Material and home environment needs	548	2.18 (0.54)	439	2.17 (0.57)	-0.01 (-0.08, 0.06)	0.77	0.004
Transport and school environment needs	556	1.66 (0.82)	434	1.78 (0.77)	0.13 (-0.02, 0.28)	0.082	0.069
Material reliability concerns	561	1.93 (0.86)	450	1.78 (0.95)	-0.13 (-0.27, 0.01)	0.070	0.034
Change and disposal insecurity	556	1.68 (0.60)	440	1.56 (0.65)	-0.10 (-0.22, 0.01)	0.083	0.072
Reuse needs	522	1.69 (1.23)	418	1.95 (1.07)	0.20 (-0.09, 0.49)	0.18	0.18
Reuse insecurity	525	2.15 (1.01)	421	1.90 (1.07)	-0.23 (-0.45, -0.01)	0.036	0.10
	Schools	Mean score (SD)	Schools	Mean score (SD)	aMD (95% CI)	p-value	
Change in gender parity in enrolment (0–2)	30	0.031 (0.088)	30	-0.004 (0.058)	-0.04 (-0.07, 0.004)	0.075	

Note: Analyses include all eligible children with outcome data at the relevant follow-up. For repeated measures, we calculated a proportion of available follow-ups with illness / absent for each participant, and the mean and SD of these proportions across participants are shown. Analyses were adjusted for stratification variables (school size < or ≥1200 pupils, and presence of a kindergarten). Except for gender parity in enrolment (measured at the school level), all analyses

included a random effect for school, and analyses based on repeated measures included an additional random effect for pupil-level clustering and assumed a constant treatment effect across time-points.

Table S5. Sensitivity analyses.

	Primary analysis (adjusted for stratification variables)		Further adjusted for responsibility for household income		Primary analysis with no age restrictions	
Outcome	aOR (95% CI)	p-value	aOR (95% CI)	p-value	aOR (95% CI)	p-value
Pupil-reported diarrhoea in past 7 days†	1.15 (0.83, 1.59)	0.39	1.16 (0.84, 1.60)	0.37	1.16 (0.84, 1.59)	0.37
Pupil-reported diarrhoea in past 2 days	1.22 (0.82, 1.83)	0.32	1.23 (0.82, 1.83)	0.31	1.23 (0.83, 1.83)	0.30
Pupil-reported respiratory illness in past 7 days†	0.84 (0.71, 1.00)	0.046	0.84 (0.71, 1.00)	0.044	0.84 (0.70, 0.99)	0.039
Pupil-reported respiratory illness in past 2 days	0.88 (0.73, 1.06)	0.18	0.88 (0.74, 1.06)	0.18	0.88 (0.73, 1.05)	0.16
	aOR (95% CI)	p-value	aOR (95% CI)	p-value	aOR (95% CI)	p-value
Roll-call absence†	1.07 (0.83, 1.38)	0.59	1.07 (0.84, 1.38)	0.58	1.04 (0.81, 1.34)	0.74
	aOR (95% CI)	p-value	aOR (95% CI)	p-value	aOR (95% CI)	p-value
Pupil-reported full-day absence in past week	1.01 (0.75, 1.36)	0.93	1.01 (0.76, 1.36)	0.92	1.00 (0.75, 1.34)	0.99
	aMD (95% CI)	p-value	aMD (95% CI)	p-value	aMD (95% CI)	p-value
SDQ-15 total difficulties score (0–40)	0.03 (-0.62, 0.68)	0.94	0.03 (-0.62, 0.68)	0.93	0.05 (-0.61, 0.71)	0.88
SAMNS-26 total score (0–100)	3.32 (0.05, 6.59)	0.046	3.34 (0.07, 6.61)	0.045	3.17 (0.006, 6.34)	0.050
MPNS-36 total score (0–3)	-0.01 (-0.08, 0.06)	0.81	-0.01 (-0.08, 0.06)	0.75	-0.003 (-0.07, 0.07)	0.94

Note: Analyses include all eligible children with outcome data at the relevant follow-up. Stratification variables adjusted for in all models are school size (< or ≥1200 pupils) and presence of a kindergarten. All analyses included a random effect for school, and analyses based on repeated measures included an additional random effect for pupil-level clustering and assumed a constant treatment effect across time-points. †Primary outcomes.
